# Pain Inhibits GRPR Neurons via GABAergic Signaling in the Spinal Cord

**DOI:** 10.1038/s41598-019-52316-0

**Published:** 2019-11-01

**Authors:** Rita Bardoni, Kai-Feng Shen, Hui Li, Joseph Jeffry, Devin M. Barry, Antonella Comitato, Yun-Qing Li, Zhou-Feng Chen

**Affiliations:** 1 Center for the Study of Itch, Washington University School of Medicine, St. Louis, MO 63110 USA; 2 Departments of Anesthesiology, Washington University School of Medicine, St. Louis, MO 63110 USA; 3 Departments of Psychiatry, Washington University School of Medicine, St. Louis, MO 63110 USA; 4 Departments of Developmental Biology, Washington University School of Medicine, St. Louis, MO 63110 USA; 5 Departments of Biomedical, Metabolic and Neural Sciences, University of Modena and Reggio Emilia, Modena, 41125 Italy; 6 Department of Anatomy & K. K. Leung Brain Research Centre, The Fourth Military Medical University, Xi’an, 710032 P.R. China; 7 Present Address: Department of Neurosurgery, Xinqiao Hospital, Third Military Medical University, Chongqing, 400037 P.R. China; 8 Departments of Life Sciences, University of Modena and Reggio Emilia, Modena, 41125 Italy

**Keywords:** Neuroscience, Social behaviour

## Abstract

It has been known that algogens and cooling could inhibit itch sensation; however, the underlying molecular and neural mechanisms remain poorly understood. Here, we show that the spinal neurons expressing gastrin releasing peptide receptor (GRPR) primarily comprise excitatory interneurons that receive direct and indirect inputs from C and Aδ fibers and form contacts with projection neurons expressing the neurokinin 1 receptor (NK1R). Importantly, we show that noxious or cooling agents inhibit the activity of GRPR neurons via GABAergic signaling. By contrast, capsaicin, which evokes a mix of itch and pain sensations, enhances both excitatory and inhibitory spontaneous synaptic transmission onto GRPR neurons. These data strengthen the role of GRPR neurons as a key circuit for itch transmission and illustrate a spinal mechanism whereby pain inhibits itch by suppressing the function of GRPR neurons.

## Introduction

Pain and itch information is conveyed by distinct yet interacting neuronal pathways in sensory neurons and spinal cord to the brain^1–6^. GRPR is a member of the mammalian homologs of the bombesin-receptor family and plays an important role in a number of physiological functions, including itch sensation^1,7,8^. GRPR and GRPR neurons in the superficial dorsal horn of the spinal cord are activated by GRP from sensory neurons to transmit itch information from the skin to the spinal cord^1,8–14^. GRPR neurons are subject to a variety of regulations, including Tac1 neurons in periaqueductal gray-mediated descending regulation^15^. GRPR can cross-talk with other G protein coupled receptors, such as MOR1D, an isoform of mu opioid receptor^8^, or 5HT1A, a serotonin receptor^16^, KOR, a kappa opioid receptor^12^, resulting in activation (MOR1D), facilitation (5HT1A) or attenuation (KOR) of the activity of GRPR neurons. GRPR neurons may form contacts with NK1R neurons which project to the parabrachial nucleus (PBN) and the spinothalamic track (STT) neurons to relay itch information in mice^13,17–19^. Despite recent progress on understanding of GRPR neuronal properties^13,20^, detailed characterization of molecular, anatomical and electrophysiological properties of GRPR neurons is yet to be achieved.

The pain pathway can suppress itch transmission as shown by empirical evidence and experimental studies^21–23^. In addition, cooling (e.g. menthol), can also relieve itch via transient receptor potential cation channel subfamily M member 8 (TRPM8) in DRG neurons^24,25^. It has been suggested that pain inhibits chemical itch through Dynorphin (Dyn) or Dyn-expressing GABAergic neurons, or BI-5 neurons, in the spinal cord^26^. While Dyn is a potent anti-itch peptide when applied exogenously, its endogenous role of Dyn or Dyn neurons in itch inhibition remains to be determined since ablation of neither Dyn nor Dyn neurons affects itch transmission^12,26,27^. In addition, activation of neuropeptide Y 1 (NPY-Y1) has recently been shown to inhibit mechanical itch^28^ and chemical itch^29^. However, whether painful stimuli can activate NPY-Y1 remains unclear.

In this study, we postulated that counter-stimuli inhibit itch by GABAergic signaling-mediated inhibition of GRPR function. We used a combination of anatomical tracing and electrophysiology to characterize the properties of GRPR neurons. Our studies reveal previously unknown features of GRPR neurons regarding their synaptic connectivity and inhibitory effects of counter-stimuli.

## Results

### GRPR neurons in the dorsal horn and SpVc are interneurons and form synaptic contacts with projection neurons

**Figure 1:**
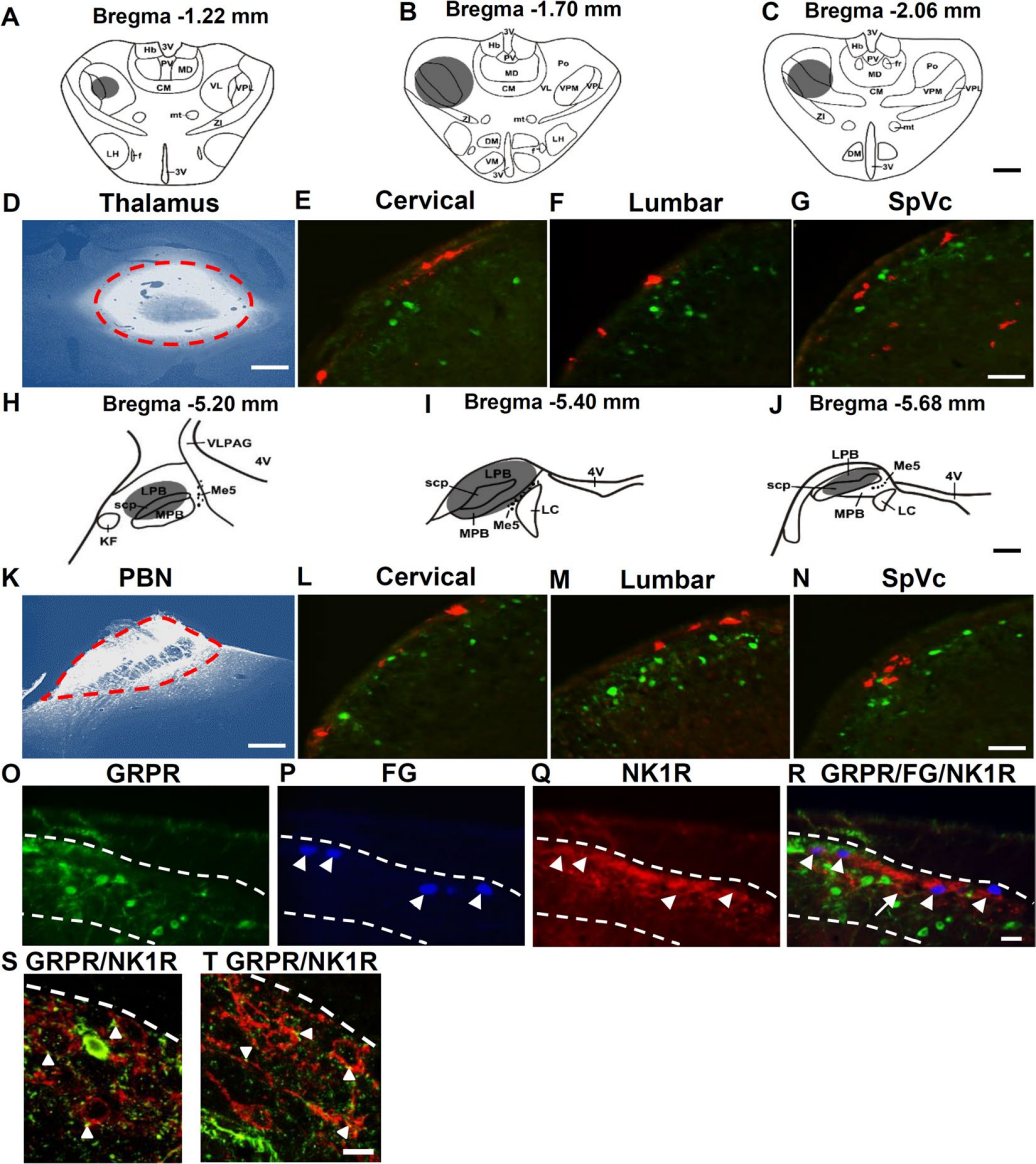
GRPR^+^ neurons in the spinal cord dorsal horn and SpVc are interneurons. (**A**–**C**) Diagrams show FG injection sites (grayed areas) in the thalamus. (**D**) FG (bright white) injection site in the thalamus was circled in red dashed line. (**E**–**G**) There was no GRPR (GFP, green) and FG (red) double-labeled cells in the dorsal horn of the cervical spinal cord **(E)**, lumbar spinal cord **(F)** and SpVc **(G)** in GRPR-eGFP mice. (**H**–**J**) the grayed areas indicate the injection site **(H)** and diffused regions **(I,J)** of FG after PBN injection. **K**, Red dashed line defines the border of injection site of FG in PBN. (**L**–**N**) Double staining in the dorsal horns of cervical spinal cord **(L)**, lumbar spinal cord **(M)** and SpVc **(N)** in GRPR-eGFP mice showed that GRPR (GFP, green) neurons were not FG (red) projection neurons to PBN. (**O**–**R**) GRPR neurons **(O)** were not co-labeled with FG (**P**, arrowheads), NK1R (**Q**, arrowheads), and FG/NK1R double-labeled neurons (**R**, arrowheads). (**S,T**) GRPR terminals (green) make contacts (arrowheads) with NK1R neurons (red) in lamina I of spinal dorsal horn. Scale bars, 600 µm in **A**–**D**,**H**–**K**; 25 µm in **E**–**G**,**L**–**R**; 10 µm in **S**,**T**.

To directly test whether GRPR neurons are interneurons or projection neurons, we performed retrograde tracing of projection neurons by injecting the retrograde fluorescent *tracer* Fluoro-Gold (FG) into the thalamus or PBN of GRPR-eGFP mice followed by double immunohistochemistry (IHC) staining as described (Fig. 1A–D,H–K)^30^. GRPR neurons are mainly distributed in laminae I and II (Fig. 1, green)^30^. Although not all GRPR neurons express eGFP, our previous studies found that all eGFP neurons analyzed express GRPR as validated by single cell RT-PCR and their responses to GRP^16^. These eGFP neurons were primarily located in the superficial dorsal horn (laminae I-II), both medially and laterally^16^. FG-labeled lamina I neurons were found predominantly in the spinal trigeminal nucleus caudalis (SpVc) and upper cervical segments of the spinal cord after FG injection into the thalamus (Fig. 1E–G, red), while after PBN injection, the majority of FG neurons were found in lumbar segments (Fig. 1I–N, red)^30^. Of 150 sections examined from different segments of the spinal cords and SpVc of mice (n = 15) that were injected with FG into thalamus or PBN, none of the eGFP neurons were co-labeled with FG. Consistent with the fact that the majority of NK1R neurons are PBN-projecting neurons in mice^17^, eGFP was not co-labeled with NK1R nor with NK1R/FG double-labeled neurons (Fig. 1O–R). However, numerous eGFP contacts were observed with NK1R neurons, suggesting that itch information from GRPR neurons is transmitted in part through NK1R neurons (Fig. 1S,T).

We next examined whether GRPR neurons form direct connection with PBN projecting neurons using double immuno-electron microscopy (Immuno-EM) for GRPR and FG in the lumbar cord. Terminals of GRPR neurons identified by the silver enhanced nanogold particles formed asymmetric synapses with FG dendritic profiles revealed by the immunoperoxidase reaction product (Supplementary Fig. 1).

### Characterization of GRPR neuron membrane properties

**Figure 2:**
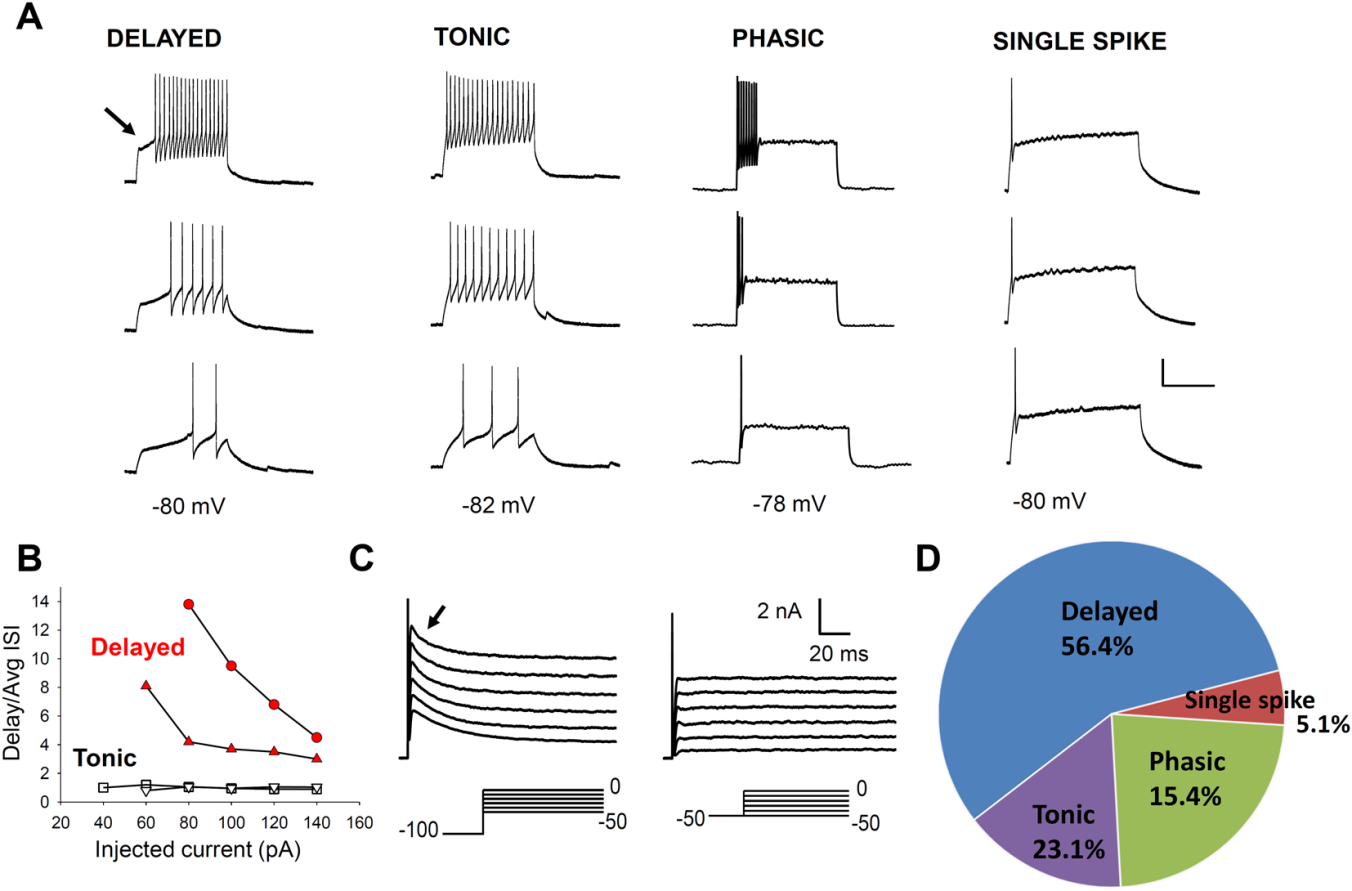
Discharge patterns observed in GRPR neurons. (**A**) Sample current clamp recordings obtained from GRPR neurons, by holding the membrane potential around −80 mV. The 3 traces for each firing type represent (starting from the lower trace): response to the first current step able to induce action potentials (rheobase) and responses to 2 stimuli above threshold. Scale Bar: 30 mV, 200 ms. (**B**) Graph representing the criterion used to discriminate between delayed and tonic firing neurons. Delayed firing neurons showed a delay to the 1st spike (indicated by the arrow in **A**) that was disproportionately long compared with the average interval between spikes (Avg ISI), even at large depolarizing current steps (red symbols, example of 2 neurons). Tonic firing neurons had delays to the 1st spike comparable to the average ISI (white symbols, 2 neurons). (**C**) Example of voltage clamp recordings obtained from a delayed firing GRPR neuron, in the presence of 1 µM TTX. When the neuron was held at −100 mV, voltage steps from −50 to 0 mV evoked a transient, potassium A-type current (marked by the arrow). When the same neuron was held at −50 mV, the A current was absent. (**D**) Proportions of neurons exhibiting the different firing types, from a total sample of 39 GRPR neurons.

To characterize the properties of GRPR neurons, electrophysiological recordings were obtained from a total of 230 GRPR-eGFP neurons in the spinal cord slices from P16-P25 mice. Action potential firing patterns were determined from a sample of 39 GRPR neurons, by recording, in current clamp, the responses to injections of depolarizing current. Most neurons (56.4%) exhibited a delayed firing pattern, when current steps were applied from a membrane potential of about −80 mV (Fig. 2A–D). This pattern is characterized by a delay in the generation of the first action potential, that is larger than the average interspike interval (Fig. 2B). Other subpopulations of GRPR neurons showed a tonic (23.1%) or a phasic (15.4%) firing pattern (Fig. 2A–D). The tonic pattern is characterized by an action potential discharge that persists during the whole current step and often decreases in frequency. The delay of the first action potential is comparable to the average interspike interval (Fig. 2B). Neurons showing the phasic pattern fire only at the beginning of the current step, with a variable number of action potentials. Only two neurons exhibited a single spike pattern. Similar results have been recently reported^31^, showing a prevalence of the delayed firing pattern in GRPR neurons recorded at their resting potential (about −73 mV).

A recent study showed that GRPR neurons predominantly exhibit a tonic firing pattern at their resting potential^20^. Accordingly, almost half of the delayed firing neurons at −80 mV exhibited a tonic pattern when maintained in current clamp at −60/−65 mV (Supplementary Fig. 2). Voltage clamp recordings performed from delayed firing neurons showed the presence of a transient, voltage-dependent A current, that was activated by holding the cells at negative potentials (−100 mV) and applying depolarizing voltage steps (Fig. 2C). Activation of the A current is likely responsible for the delayed firing pattern observed in most GRPR neurons at −80 mV, as shown in previous studies^32,33^.

To confirm the expression and functionality of GRPR on GRPR neurons, we tested the response of 22 neurons to GRP. Application of 1 µM of GRP induced a slow inward current in 73% (16/22) of GRPR neurons, held at −50 mV in voltage clamp. A second application of GRP, performed on a subpopulation of 7 responsive neurons, elicited in 3 cells an inward current of smaller amplitude (11.5 ± 6.8 pA versus 22.6 ± 10.1 pA at the first GRP application). The suppression of an inward rectifier K^+^ current and/or the activation of a non-selective cation conductance could contribute to the GRP-generated current (Supplementary Fig. 3)^34^.

### GRPR neurons receive direct inputs from primary afferents

**Figure 3:**
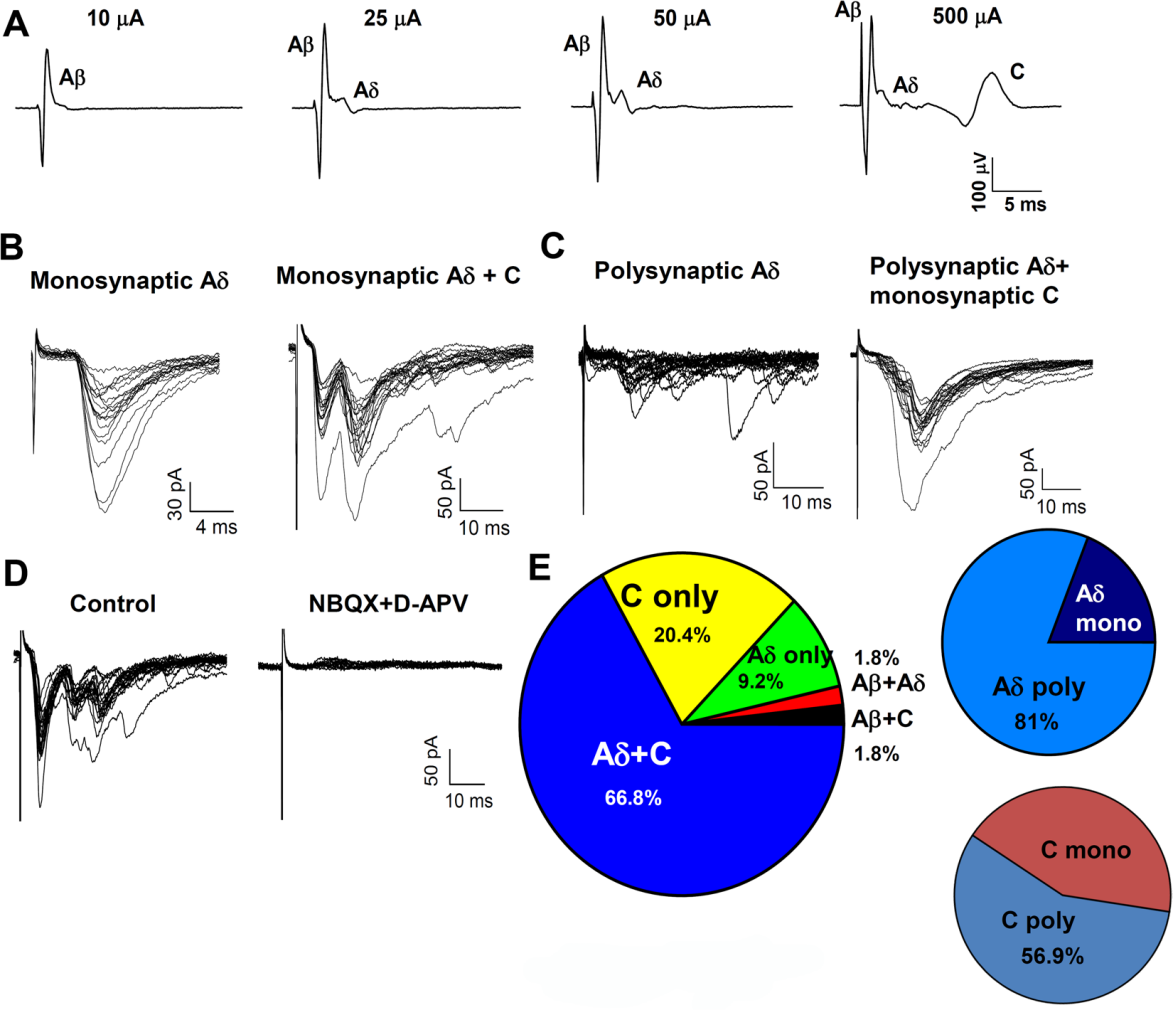
Characterization of excitatory synaptic inputs to GRPR neurons. (**A**) Compound action potentials (CAPs) recorded from a lumbar dorsal root of a *GRPR-*eGFP mouse. CAPs were evoked by stimulating the dorsal root with a suction electrode at different stimulus intensities. Labelling indicates the voltage peaks corresponding to the different afferent fibers. (**B**) Example of evoked EPSCs recorded from a GRPR neuron, at different stimulus intensities and frequencies. An Aδ monosynaptic input is evoked at 50 µA, showing no failures and constant latency at 20 Hz. By stimulating at 500 µA and 1 Hz, a monosynaptic C input becomes apparent. (**C**) Example of evoked EPSCs, recorded from a different GRPR neuron, using a similar protocol as in B. This neuron received a polysynaptic Aδ input, that failed at 2 or 10 Hz. By stimulating at higher intensities (500 µA) a monosynaptic C input was recruited, showing no failures at 1 Hz. (**D**) Evoked EPSCs were mediated by glutamate ionotropic receptors (AMPA and NMDA), since they were completely blocked by 10 µM NBQX and 50 µM D-APV (*n* = 10). (**E**) Characterization of the primary afferent inputs mediating evoked EPSCs in GRPR neurons (*n* = 54). In most neurons, EPSCs were mediated by both Aδ and C fibers. Polysynaptic inputs were predominant for both Aδ and C fibers. Only a very small proportion of synapses could be classified as Aβ-mediated (all polysynaptic).

Previous studies showed that abundant GRP fibers are present in the dorsal horn^9,35,36^ and Immuno-EM studies confirmed that GRP fibers form contacts with dendrites of GRPR neurons^37^. To characterize the primary afferent fibers synapsing onto GRPR neurons, we stimulated the dorsal root attached to the slice and recorded evoked excitatory postsynaptic currents (EPSCs) from GRPR neurons (Fig. 3). Stimulus intensities were determined in separate sets of experiments, by stimulating one end of the dorsal root and recording compound action potentials from the other end (n = 8, Fig. 3A). Intensities of stimulation ranged between 10 and 25 μA for Aβ fibers, 25 and 100 μA for Aδ fibers, and between 200 and 500 μA for C fibers. These values are consistent with those reported by previous studies performed in mice of comparable age^38^.

**Figure 4:**
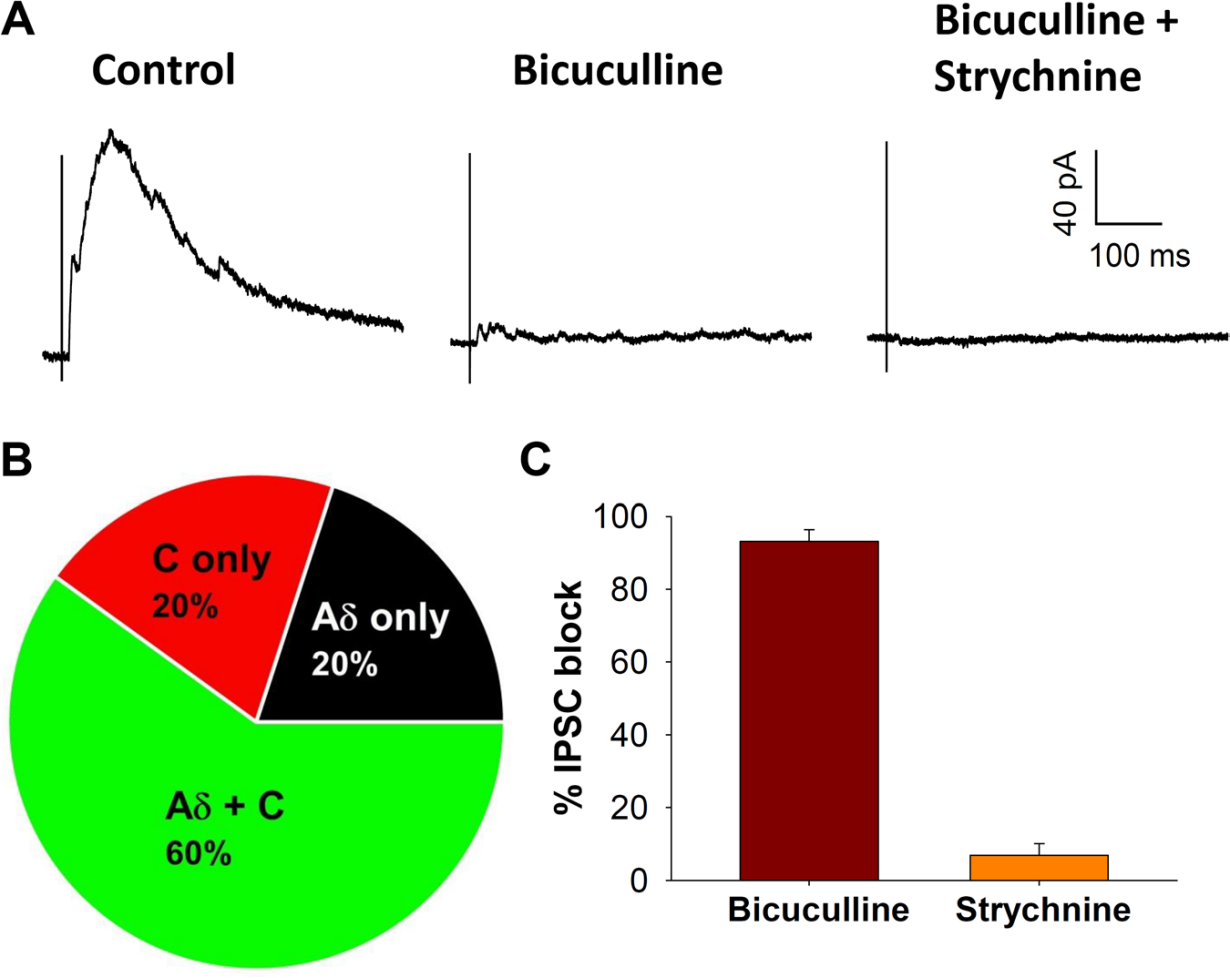
Characterization of inhibitory synaptic inputs to GRPR neurons. (**A**) Example of evoked IPSCs recorded from a GRPR neuron held at −10 mV. The IPSC in control shows a faster component mediated by Aδ fibers (evoked by stimulating at 50 µA) and a slower current, mediated by C fibers (evoked at 500 µA). 10 µM bicuculline blocked almost completely the IPSC. Application of bicuculline plus 300 nM strychnine caused a complete block. (**B**) Characterization of the primary afferent inputs mediating evoked IPSCs in GRPR neurons (*n* = 25). In most neurons, IPSCs were mediated by both Aδ and C fibers. (**C**) Percentage block of the evoked IPSCs, observed in presence of 10 µM bicuculline and 300 nM strychnine (*n* = 10). GABA_A_ receptors mediate most of the IPSCs recorded from GRPR neurons. Data are represented as mean ± SEM.

Of 54 GRPR neurons, the majority of cells received synaptic input from both Aδ and C fibers (n = 36, 66.8%), while a small proportion exhibited EPSCs mediated by Aδ or C fibers only (n = 11 and 5, respectively; Fig. 3B,C,E). EPSCs mediated by Aδ were mostly polysynaptic (n = 38, 81%), while almost half of the C fiber mediated responses were monosynaptic (n = 18, 43.1%). Aβ fiber mediated EPSCs (of polysynaptic nature), evoked at intensities lower than 25 µA, were observed in only 2 of the 54 neurons tested, indicating that GRPR neurons mainly receive primary afferent inputs recruited by high intensity stimulation. Other 4 cells, showing polysynaptic EPSCs evoked at 25 µA, were classified as receiving Aδ inputs, although at this stimulus intensity some additional Aβ fibers could have been also recruited (Fig. 3A). Being polysynaptic connections, it was not possible to apply the high frequency stimulation protocol to distinguish the 2 types of fibers. EPSCs evoked on GRPR neurons by dorsal root stimulation were completely blocked by co-application of the AMPA receptor antagonist NBQX and the NMDA receptor antagonist D-APV, showing that they are mediated by glutamate receptors (n = 10, Fig. 3D). Stimulation of Aδ and C fibers was also able to evoke inhibitory postsynaptic currents (IPSCs) on GRPR neurons held at −10 mV (Fig. 4A). Similar to the EPSCs, most neurons exhibited evoked IPSCs mediated by both Aδ and C fibers (15 out of 25 neurons tested, Fig. 4B). Neurons showing IPSCs mediated by Aδ fibers include also 3 cells where IPSCs were evoked at 25 µA, an intensity also compatible with Aβ stimulation (see above). Application of bicuculline produced more than a 90% block of the evoked IPSCs, showing that they were mainly mediated by GABA_A_ receptors (n = 10, Fig. 4C).

### Inhibition of GRPR neurons by pain and cooling

**Figure 5:**
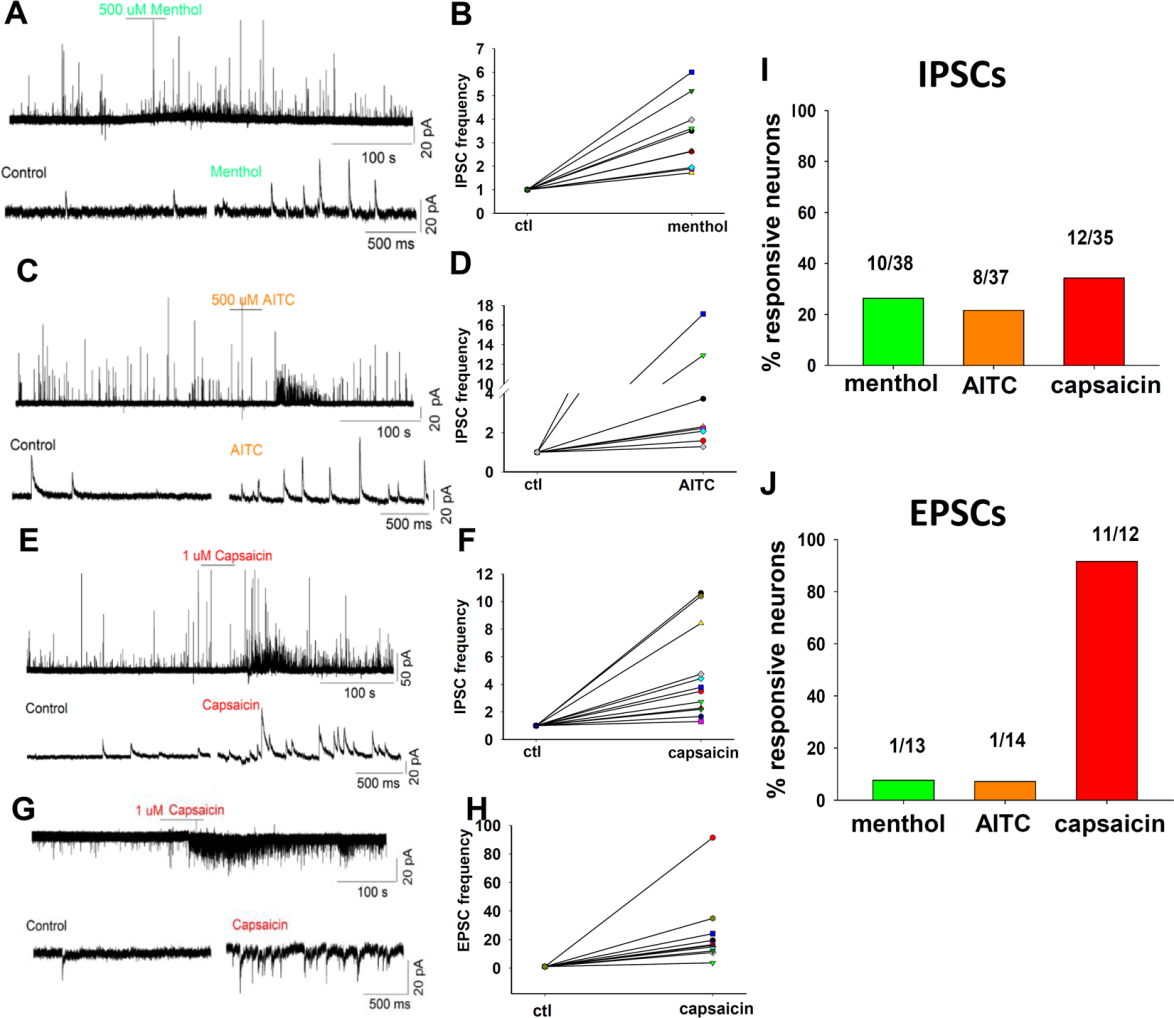
Counter-stimuli increase inhibition onto GRPR neurons. (**A,C,E**) Example traces of spontaneous IPSCs recording from GRPR neurons, held at −10 mV. One min application of 500 µM menthol (**A**), 500 µM AITC (**C**) or 1 µM Capsaicin (**E**) produced a significant increase of sIPSC frequency in subpopulations of GRPR neurons. Statistical significance (*p* < 0.05) was determined by using the Kolmogorov-Smirnov test on individual neurons. Lower traces depict sIPSCs on an expanded time scale for control and counter-stimuli applications. (**B,D,F**) Normalized sIPSC frequencies observed in the responsive neurons in the presence of the counter-stimuli. (**G**) Application of capsaicin induced also a strong frequency increase of sEPSCs, recorded at – 60 mV. (**H**) Scatter plot of normalized sEPSC frequencies, obtained from a sample of GRPR neurons responsive to capsaicin. (**I,J**) Percentages of GRPR neurons exhibiting an increase of sIPSC or sEPSC frequency upon application of the 3 counter-stimuli. Data are represented as mean ± SEM.

The observation that evoked IPSCs on GRPR neurons are mediated by primary afferents stimulated at high intensity suggests that these neurons are inhibited by nociceptive inputs. To test this hypothesis, we examined the effect of capsaicin, allyl isothiocyanate (AITC, a key component of mustard oil) and menthol on spontaneous IPSCs (sIPSCs) recorded from GRPR neurons. Upon application of menthol (500 µM), we observed a significant increase of sIPSC frequency in 26.3% of GRPR neurons, with an average 3.3-fold increase (Fig. 5A,B,I). AITC (500 µM) significantly increased the sIPSC frequency in 21.6% of neurons, with an average 5.3-fold increase (Fig. 5C,D,I). An increase of sEPSC frequency was observed in one neuron out of 13 in menthol and in one neuron out of 14 in AITC (Fig. 5J). Remarkably, application of capsaicin (1 µM) affected both sEPSCs and sIPSCs of GRPR neurons (Fig. 5E–J), with a potent effect (23.5-fold increase) on sEPSC frequency in the large majority of cells tested (91.6%). By contrast, sIPSC frequency was increased 4.6-fold in 34.3% of the recorded neurons. These results confirm that pain and cooling can activate inhibitory spinal interneurons, producing an increase of the inhibitory tone in GRPR neurons.

## Discussion

In this study, using classic neuroanatomical tracing and immuno-EM approach we demonstrate that GRPR neurons are excitatory interneurons that make contacts with NK1R PBN- and STT-projecting neurons, supporting earlier studies^13,14^. Electrophysiological studies show that the majority of GRPR neurons exhibit a delayed firing pattern at hyperpolarized potentials, typical of dorsal horn excitatory interneurons^33,39^, which is consistent with the study by Aresh *et al*.^20^, showing that the majority of GRPR neurons express *Vglut2* mRNA.

Importantly, we have characterized, for the first time, the primary afferent fibers mediating excitatory and inhibitory synaptic transmission onto GRPR neurons. GRPR neurons receive both direct and indirect high threshold C/Aδ excitatory inputs from primary afferents, in line with earlier studies in primates and humans showing that primary afferent pruriceptors are high threshold C/A fibers^40–43^. However, it is surprising that we failed to find significant Aβ inputs onto GRPR neurons using transverse spinal cord slices, a preparation that has been shown to be suitable for studying mono- and polysynaptic responses mediated by Aβ fibers^44,45^. However, it is possible that synaptic circuits activating superficial GRPR neurons (sometimes selected because of the stronger fluorescent signal) were not preserved in our preparation, thereby contributing to an underestimation of the amount of Aβ inputs received by GRPR neurons in the present study. The fact that EPSCs evoked by the dorsal root stimulation were blocked by NBQX and D-APV demonstrates that fast glutamatergic transmission constitutes an integral mechanism for relaying itch information from primary afferents to GRPR neurons. While a high concentration of GRP can directly evoke spikes on GRPR neurons^20^, at a lower dose that is of more physiological relevance, GRP could only depolarize cells, suggesting that GRP *in vivo* may modulate glutamatergic transmission under normal physiological condition^16,46^. GRP may modulate an indirect glutamatergic transmission from NMBR neurons to GRPR neurons^46^, rather than primary afferent-dependent glutamatergic input, which is not required for nonhistaminergic itch transmission^47,48^. It is worth noting that spinal neurons expressing *Grp* mRNA has been suggested to be important in itch transmission through activation of natriuretic peptide receptor A (NPRA) by B-type natriuretic peptide released from sensory neurons^49^. Using intersectional spinal restricted ablation of *Grp* neurons, we found that spinal *Grp* neurons are dispensable for itch transmission^12^. Equally important is the finding that conditional knockout of *Grp* in sensory neurons revealed the deficit in nonhistaminergic but not histamine itch^12^, consistent with earlier studies^9,10,46^.

Another novel finding is that inhibitory synaptic transmission onto GRPR neurons is mediated by high threshold Aδ/C fibers. The observation that over 90% of evoked IPSCs were blocked by bicuculline indicates that fast GABAergic transmission is necessary and sufficient to mediate inhibition of GRPR neurons. These results are consistent with recent studies showing anti-itch effects of GABA, a major neurotransmitter to inhibit laminae I-II excitatory interneurons located by GABA^50^, in various itch conditions^51,52^. One important question arising from the present study is which subset of inhibitory neurons release GABA to inhibit GRPR neurons. One candidate is SST2A inhibitory neurons whose inhibition by exogenous somatostatin (SST) may result in disinhibition of itch transmission^26,53,54^. It is possible that SST2A interneurons could be activated by painful stimuli, since mustard oil, capsaicin and menthol increase glutamatergic transmission on these neurons^26^.

By directly recording the responsiveness of GRPR neurons, we show that the same counterstimuli increase spontaneous inhibitory currents on many GRPR neurons. Thus, our data represent the first evidence that GABAergic interneurons, innervated by sensory neurons responsive to counterstimuli, are synaptically connected to GRPR neurons. On the other hand, since SST2A neurons are also required for pain inhibition and i.t. SST-elicited scratching reflects both itch and pain, the question remains as to whether there is dedicated neuronal pathway for itch inhibition. In addition, future studies are necessary to determine which GABA subtype receptors are expressed in GRPR neurons^55^.

Peripherally administered mustard oil (AITC) can induce both pain and itch in a dose-dependent manner^56^. Application of AITC on lamina II neurons in slices potentiates both spontaneous and miniature EPSCs^57^. In our experiments, AITC seems to act predominantly on nociceptive afferents, increasing inhibition on GRPR neurons. Excitatory responses recorded from GRPR neurons were not significantly affected by AITC, suggesting a lack of effect of mustard oil on central terminals of pruriceptive fibers. Topical application of capsaicin can induce a mix of pain and itch sensations^58,59^. Interestingly, we found that capsaicin exhibited dual effect on GRPR neurons: while inhibiting some, it also activates GRPR neurons. These results suggest that capsaicin induces pain by activating non-MrgprA3 TRPV1 nociceptors^60^, which in turns inhibits GRPR neurons, while concurrently inducing itch by activating TRPV1 in pruriceptors^61^ and/or MrgprA3 neurons, which mediates itch via innervating GRPR neurons^60^. Coupled with previous studies, GRPR neurons receive at least three different kinds of inputs from primary afferents: a direct input from C/Aδ pruriceptors, an indirect glutamatergic input from NMBR neurons^30^, and an indirect inhibitory input from GABAergic neurons, in part mediated by Vglut2-expressing primary afferents^47,48^. Therefore, there are several modes of action of GRPR neurons, whose activation or inhibition could underlie the responses of itch behavior mediated by various counter-stimuli.

## Methods

### Animals

Experiments were carried out on C57BL/6 J mice and GRPR-eGFP mice (Stock no. 036178-UCD, MMRRC). All mice were housed under a 12 h light/dark cycle. Mice were housed in clear plastic cages with no more than 5 mice per cage in a controlled environment at a constant temperature of ~23 °C and humidity of 50 ± 10% with food and water available *ad libitum*. All experiments conform to guidelines set by the National Institutes of Health and the International Association for the Study of Pain and were reviewed and approved by the Animal Studies Committee at Washington University School of Medicine. The Italian Ministry of Health approved all electrophysiology experiments in accordance with the Guide for the Care and Use of Laboratory Animals and the EU and Italian regulations on animal welfare.

### Retrograde tracing

Retrograde tracing was performed as described previously^30^. GRPR-eGFP male mice were anesthetized with an intraperitoneal injection of ketamine/xylazine (90 mg/kg; 10 mg/kg) cocktail, injected with buprenorphine (BupSR, 0.5 mg/kg) for analgesia, and placed onto a stereotaxic frame (Stoelting, Wood Dale, IL, USA). A volume of 0.15~0.25 µl of 4% FG (Biotium, Hayward, CA, USA) was filled into a glass pipette (internal tip diameter 15–20 µm) attatched to a Nanoject II auto-nanoliter injector and was injected into each injection site. For thalamus (ventral posterolateral thalamic nucleus-VPL, ventral posteromedial thalamic nucleus-VPM, posterior thalamic nuclear group-Po, posterior thalamic nuclear group, triangular part-PoT), 0.15 µl FG was injected into site a (AP −1.22, ML ± 1.45, DV −3.30), 0.25 µl into site b (AP −1.70, ML ± 1.60, DV −3.40), and 0.15 µl into site c (AP −2.06, ML ± 1.40, DV −3.30). For parabrachial nucleus (PB), 0.25 µl of FG was injected into one site (AP −5.20, ML ± 1.25, DV −2.40). 7 days after surgery, mice were anesthetized with an overdose of ketamine/xylazine cocktail and perfused with 0.1 M PBS and then 4% paraformaldehyde. The brain and spinal cord were removed, post-fixed in the same fixative for 6 h, cryoprotected overnight in 30% sucrose in PBS. Brains and spinal cords were sectioned into 50 µm and 20 µm thick sections, respectively, for injection sites observation or immunofluorescent staining.

### Immunohistochemistry

The procedures were described previously^37,62^. Deeply anesthetized mice (ketamine, 90 mg/kg and Xylazine, 10 mg/kg) were perfused transcardially with 0.01 M PBS (PH 7.4) and paraformaldehyde (PFA) (4% in PBS). Spinal cord and brain were removed and post-fixed in 4% PFA for 2–4 h. The tissues were then cryoprotected in 20% sucrose overnight at 4 °C. Free-floating frozen sections were incubated with 2% donkey serum and 0.3% Triton X-100 for 1 h at room temperature followed by incubation with primary antibodies overnight at 4 °C. The sections were then washed and incubated with secondary antibodies for 2 h at room temperature. The following primary antibodies were used: chicken anti-GFP (1:500, Aves Labs, GFP-1020), guinea-pig anti-NK1R (1:500, AB15810, EMD Millipore), rabbit anti-FG (1:5000, AB153, Millipore). The following secondary antibodies were used: Alexa-Fluor 488 conjugated donkey anti-chicken (1:1000, Jackson ImmunoResearch, 703–545–155), Cy3-conjugated donkey anti-rabbit (1:1000, Jackson ImmunoResearch, 711–165–152) and Cy5-conjugated donkey anti-guinea pig 1(:1000, Jackson ImmunoResearch, 703–175–148). Fluorescent Images were taken using a Nikon C2+ confocal microscope system (Nikon Instruments, Inc.).

### Immuno-electron microscopy

To observe the connections between GRPR+ neurons and FG retrograde labelled PBN projection neurons in the spinal dorsal horn, immuno-electron microscopic studies were performed as previously described^37^. Briefly, for GRPR/FG double staining, cross sections of lumbar spinal cord of adult GRPR-eGFP mice were double immune-labeled by chicken anti-GFP antibody (1:500; Aves Labs) and rabbit anti-FG (1:5000, AB153, Millipore) using immunogold-silver method and immunoperoxidase method, respectively. Further, 50-nm-thick ultrathin sections were cut and examined with a JEM-1400 electron microscope (JEM, Tokyo, Japan). The digital micrographs were captured by VELETA (Olympus,Tokyo, Japan).

### Spinal cord slice preparation

Slice preparation and electrophysiological recordings were performed as previously described^45^. Briefly, *GRPR-*eGFP mice (P16-P25) were anesthetized with isoflurane and decapitated, the spinal cord and vertebrae were rapidly removed and placed in ice-cold dissecting Krebs’ solution (composition in mM:125 NaCl, 2.5 KCl, 1.25 NaH_2_PO_4_, 26 NaHCO_3_, 25 glucose, 6 MgCl_2_, 1.5 CaCl_2_, and 1 kynurenic acid, pH 7.4, 320 mOsm), bubbled with 95% O_2_, 5% CO_2_. The lumbar spinal cord was isolated, embedded in an agarose block (low melting point agarose 3%, Thermo Fisher Scientific, Waltham, USA), and transverse slices (500 µm thick) were obtained using a vibrating microtome (WPI, Sarasota, USA). Slices were incubated in oxygenated incubation Krebs’ solution (same as dissecting but without kynurenic acid) at 35 °C for 30 min and then used for recording.

### Patch-clamp recording and dorsal root stimulation

Spinal cord slices were prepared from *GRPR-*eGFP mice (P16-P25), as previously described^45^. Patch-clamp recording in whole-cell configuration was performed on visually identified fluorescent *GRPR-*eGFP neurons at room temperature^63^. Intracellular solution filling the recording electrodes contained (in mM): 120 potassium methane-sulfonate, 10 NaCl, 10 EGTA, 1 CaCl_2_, 10 HEPES, 5 ATP-Mg, pH adjusted to 7.2 with KOH, osmolarity 300 mOsm. Recordings in voltage clamp that required holding the neuron at −10 mV were performed by using an intracellular solution having the following composition (in mM): 130 cesium methane-sulfonate, 10 sodium methanesulfonate, 10 EGTA, 1 CaCl_2_, 10 HEPES, 5 lidocaine N-ethyl bromide quaternary salt-Cl, 2 ATP- Mg, pH adjusted to 7.2 with CsOH, osmolarity 300 mOsm.

Excitatory or inhibitory currents (EPSCs or IPSCs) were evoked by stimulating the dorsal root attached to each slice using a suction electrode. Stimulus duration was 0.1 ms, stimulus intensities were determined by performing extracellular recordings of compound action potentials from the dorsal root (see Results and Fig. 5A). Monosynaptic vs polysynaptic EPSCs were identified following the procedure described by Torsney and MacDermott^44^ and Daniele and MacDermott^38^. In particular, EPSCs were considered to be monosynaptic if there were no failures during high frequency stimulation (20 pulses at 20 Hz for Aβ, at 10 or 2 Hz for Aδ and 1 Hz for C fibers). Onset latency varied <1 ms for monosynaptic A fiber mediated EPSCs.

Active and passive membrane properties were determined by applying current steps (10 or 20 pA of amplitude) in current clamp, starting from a membrane potential of −60/−65 mV. Membrane resistance was calculated from responses to hyperpolarizing pulses, rheobase was defined as the current step amplitude able to elicit the lowest number of action potentials^64^. Action potential threshold was determined for the first action potential evoked at rheobase, at the point where the increase of membrane potential exceeds 20 mV/ms.

Drugs were bath-applied for 1 min. All drugs were obtained from Sigma-Aldrich (Saint Louis, USA), except for GRP, that was purchased from Genscript (Piscataway, USA), and tetrodotoxin (TTX) from Tocris (Bristol, UK). Data were analyzed off-line using pClamp10 or MiniAnalysis (Synaptosoft, Decatur, USA). Graphs were obtained using Sigmaplot 11 (Systat software, San Jose, USA).

### Experimental design and statistical analysis

Electrophysiology experiments were performed on spinal cord slices from GRPR eGFP mice of either sex. A total of 92 mice have been used. Typically, 2–3 viable slices, with well-preserved synaptic connections, were obtained from the lumbar segment of the spinal cord (L3-L5). One to three neurons were recorded from each slice (only one cell if drugs were applied). Only neurons showing stable recording conditions (constant series resistance and membrane potential) were included in this study. Sample sizes were established according to similar studies of firing pattern and synaptic input characterization^33,38^.

Statistical tests are indicated in figure legends when performed. Values are reported as the mean ± standard error of the mean (SEM). Statistical analyses were performed using Prism 7 (v7.0c, GraphPad, San Diego, CA) or Sigmaplot 11. Normality and equal variance tests were performed for all statistical analyses. *P* < 0.05 was considered statistically significant.

## Supplementary information

SUPPLEMENTARY INFO

## Data Availability

The authors declare that the data will be available without restrictions.
