# Supplementary material for: Pain Inhibits GRPR Neurons via GABAergic Signaling in the Spinal Cord
Source: Sci Rep. 2019 Nov 1;9:15804. doi: 10.1038/s41598-019-52316-0 (PMC6825123; doi:10.1038/s41598-019-52316-0)
Supplement: Supplementary file 1 — SUPPLEMENTARY INFO [file 41598_2019_52316_MOESM1_ESM.docx]

**Pain Inhibits GRPR Neurons via GABAergic Signaling in the Spinal Cord**

Rita Bardoni, Kai-Feng Shen, Hui Li, Joseph Jeffry, Devin M. Barry, Antonella Comitato, Yun-Qing Li, & Zhou-Feng Chen

**
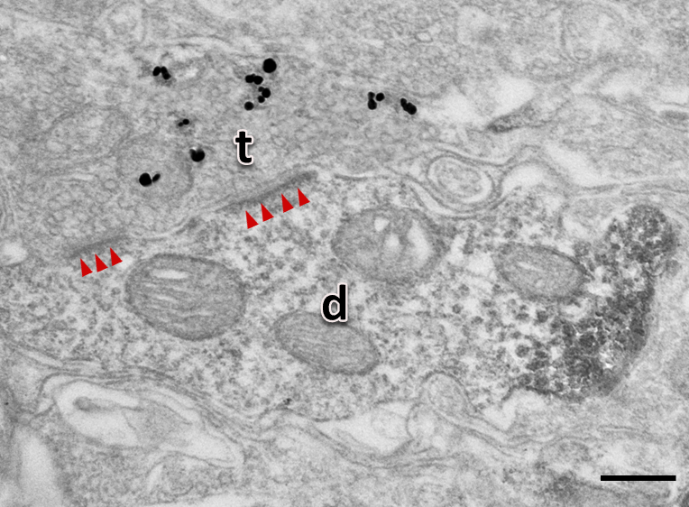
**

**Supplementary Figure 1.** GRPR fibers contacts on PBN-projecting FG retrograde labelled neurons in the superficial spinal dorsal horn. GRPR axon terminal (t; silver grains) makes asymmetric synaptic contact with FG dendritic profile (**d**; DAB reaction products). Arrow heads indicate post-synaptic membranes. Scale Bar, 0.25 µm.

**
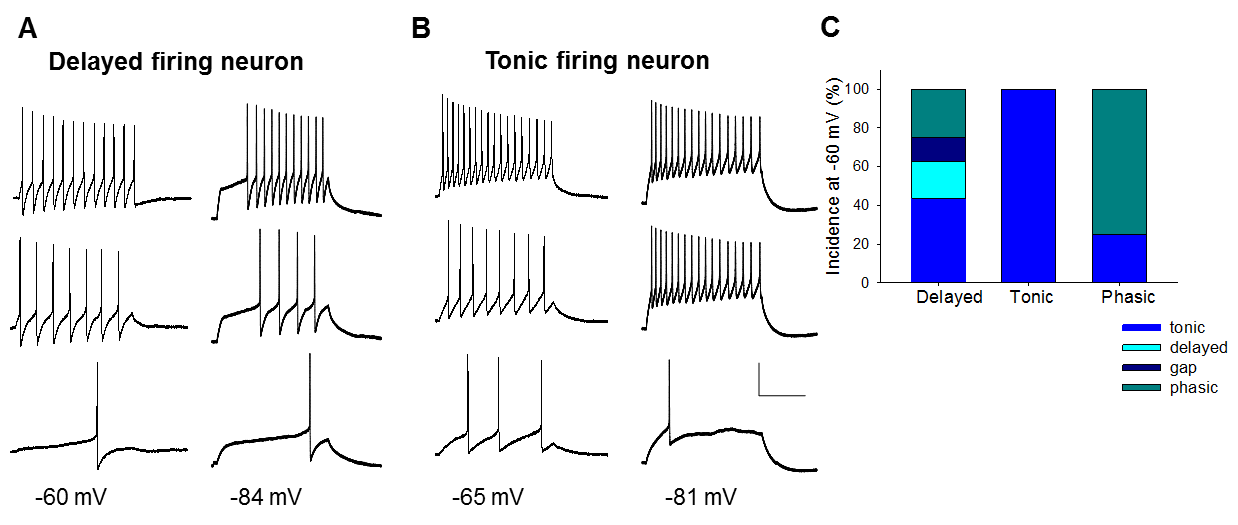
**

**Supplementary 2.** Voltage-dependence of firing patterns in GRPR neurons. (**A**) Sample current clamp recordings obtained from a neuron exhibiting a delayed firing pattern at -84 mV (right). Holding the neuron near -60 mV, a tonic pattern becomes apparent (left). The 3 traces represent (starting from the lower trace) the response to rheobase and the responses to 2 stimuli above threshold. (**B**) Sample current clamp recordings obtained from a neuron exhibiting a tonic firing pattern at -81 mV (right). A similar firing pattern is present also at -60 mV (left). Scale Bar: 30 mV, 300 ms. (**C**) Incidence of the different firing patterns at -60 mV for neurons exhibiting delayed (*n* = 16), tonic (*n* = 8) or phasic patterns (*n* = 4) at -80 mV.

**
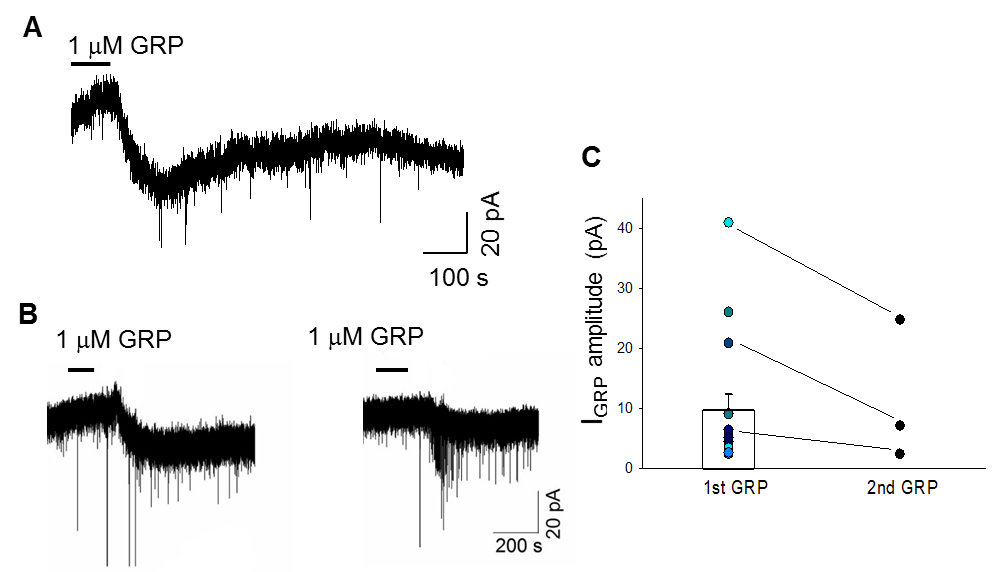
**

**Supplementary 3.** GRP application generates a slow inward current in GRPR neurons. (**A**) Example trace representing the slow inward current generated by application of 1 µM GRP onto a GRPR neuron held at -50 mV. (**B**) Currents elicited by 2 consecutive GRP applications onto a different GRPR neuron. (**C**) Amplitudes of the GRP-induced current recorded from a sample of 16 responsive GRPR neurons. Seven of these neurons were tested for a second application of GRP, that produced an inward current in 3 cells. Data are represented as mean ± SEM.


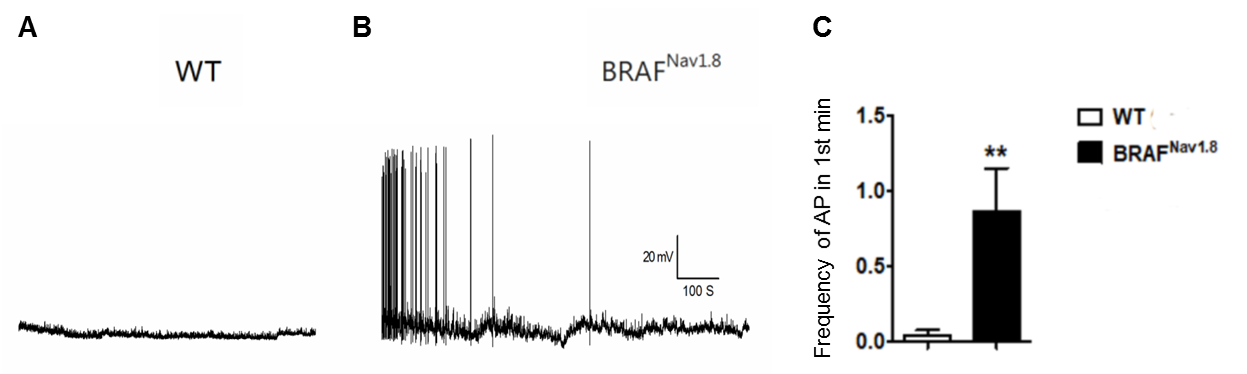


**Supplementary 4.** Increased excitability of GRPR neurons in BRAF^Nav1.8^ mice. (**A,B**) Current clamp recordings at -50/-55 mV obtained from GRPR neurons derived from w/t or BRAF^Nav1.8^ mice. Only GRPR neurons from BRAF^Nav1.8^ mice presented spontaneous, repetitive firing during the early phase of recording. (**C**) Histogram representing the average frequency of spontaneous action potentials recorded, during the first minute, from the 2 samples of GRPR neurons. Action potential frequency in GRPR neurons from BRAF^Nav1.8^ mice is significantly higher than in w/t mice (Student’s unpaired *t*-test, *p* = 0.0085, *n* = 10). Data are represented as mean ± SEM.
